# Fabrication of *in-situ* grown graphene reinforced Cu matrix composites

**DOI:** 10.1038/srep19363

**Published:** 2016-01-14

**Authors:** Yakun Chen, Xiang Zhang, Enzuo Liu, Chunnian He, Chunsheng Shi, Jiajun Li, Philip Nash, Naiqin Zhao

**Affiliations:** 1 Key Laboratory of Composite and Functional Materials, School of Materials Science and Engineering, Tianjin University, Tianjin 300072, China; 2 Collaborative Innovation Center of Chemical Science and Engineering, Tianjin University, Tianjin 300072, China; 3 Thermal Processing Technology Center, Illinois Institute of Technology, Chicago, IL 60616, USA

## Abstract

Graphene/Cu composites were fabricated through a graphene *in-situ* grown approach, which involved ball-milling of Cu powders with PMMA as solid carbon source, *in-situ* growth of graphene on flaky Cu powders and vacuum hot-press sintering. SEM and TEM characterization results indicated that graphene *in-situ* grown on Cu powders guaranteed a homogeneous dispersion and a good combination between graphene and Cu matrix, as well as the intact structure of graphene, which was beneficial to its strengthening effect. The yield strength of 244 MPa and tensile strength of 274 MPa were achieved in the composite with 0.95 wt.% graphene, which were separately 177% and 27.4% enhancement over pure Cu. Strengthening effect of *in-situ* grown graphene in the matrix was contributed to load transfer and dislocation strengthening.

Unique structure containing few layers of sp^2^ hybridized carbon atoms^1^ in a hexagonal lattice endows graphene with exceeding superior mechanical and functional properties, such as incomparable mechanical strength and Young’s modulus, extremely high thermal conductivity and charge-carrier mobility^2^,^3^,^4^,^5^. Thus, metal matrix composites (MMCs) strengthened with graphene have drawn much attention during the past few years, for it has great potential to obtain high performance to meet with the requirements of high strength, good toughness and light weight^6^,^7^,^8^,^9^,^10^,^11^. However, hanging atoms on the edges of graphene make graphene unstable and extremely likely to agglomerate or even restack to form thin carbon sheets or graphite via van der Waals force and π − π reaction^12^, resulting in great difficulties in the fabrication of MMCs.

So far, graphene used to fabricate MMCs is all *ex-situ* added to metal matrix. Most research has focused on the combination of metal powders with reduced graphite oxide (RGO) and graphene nanoplatelets (GNP) through chemical integration or mechanical integration to get a desirable dispersion of graphene within the metal matrix. For chemical integration method, electrostatic adsorption between hydrolysed Al ions and negative-charged graphite oxide (GO) has been employed to achieve the desired dispersion of GO on Al powders^13^. Jaewon Hwang *et al.*^14^ synthesized RGO/Cu composite powders by mixing GO with Cu(CH_3_COO)_2_ solution and further reduction. However, the incomplete reduction of GO and the reunion of RGO in the reduction process could influence the reinforcing effect of graphene. On the other hand, mechanical integration through mechanical ball-milling of metal powders and graphene is widely used to attain a uniform dispersion of graphene within a metal matrix. For example, Li *et al.*^15^ added RGO into Al powders and realized combination between RGO and Al powders through cryomilling. A high dispersion of graphene in a metal matrix is obtained through ball-milling of GNP with metal powders, during which the GNP was stripped and dispersed within the matrix^16^,^17^. Ball-milling is an easy and practicable method, but it introduces many defects into the graphene inevitably, which is also detrimental for its strengthening effect^5^,^6^,^7^,^14^. Therefore, although numerous works have demonstrated that MMCs could be reinforced with the addition of graphene, shortcomings in conventional methods using RGO or GNP as reinforcements directly added into the metal matrix have limited the research progress^17^,^18^,^19^. Hence, it is of great value to fabricate MMCs reinforced with *in-situ* grown graphene in the future works in this area.

Cu matrix composites exhibit a broad range of applications in different areas, such as automobiles, microelectronics and so on^20^. Traditional reinforcements used to fabricate Cu matrix composites such as oxides and carbide nanoparticles do result in significant improvement in mechanical properties of Cu^21^. Nevertheless, poor electrical and thermal conductivity of these reinforcements make them unsuitable for electronic applications. Thus, structure-intact graphene as the reinforcement for Cu composite is of great potential to fabricate a desirable Cu matrix composite. Recently, Wang *et al.*^22^ realized evenly coated PMMA on the surfaces of metal powders with the help of polyvinyl alcohol (PVA) as the binder and further obtained *in-situ* grown graphene within the metal matrix. Moreover, monolayer graphene *in-situ* grown on Cu matrix was achieved by catalyzing PMMA coated on Cu foils^4^. Thereby, graphene *in-situ* grown within a Cu matrix directly provides a good approach to overcome the bottlenecks aroused by chemical integration and mechanical integration and to achieve a good dispersion of graphene within a Cu matrix.

In this work, we introduce a favorable method to fabricate graphene/Cu composites with graphene *in-situ* grown on Cu powders from the solid carbon source PMMA, guaranteeing a good dispersion and interface between graphene and Cu matrix. After the optimization of the process parameters, bulk graphene/Cu composites are prepared and tested. Furthermore, we demonstrate the strengthening mechanisms of *in-situ* grown graphene through the characterizations of SEM and TEM along with experimental procedure. The aim of this work is to meet the ever increasing demands for structural strength and energy efficiency in the future.

Figure 1 is a brief schematic illustration of procedures to fabricate graphene/Cu composites. A detailed discussion of the sample preparation is given in the Methods section.

## Results

**Figure 2:**
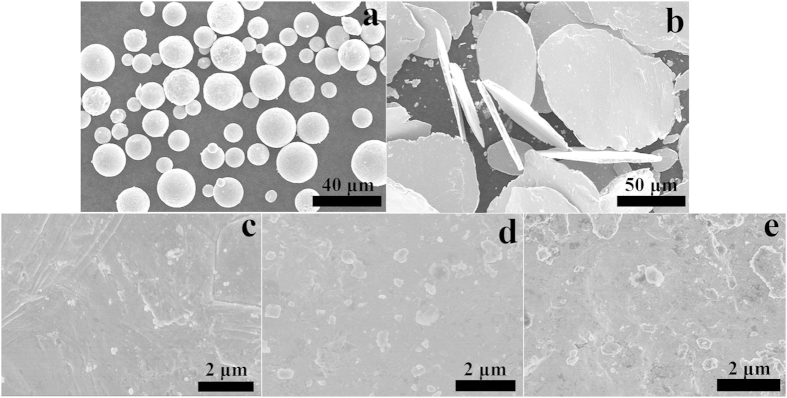
(**a**) SEM image of original pure Cu powders. (**b**) Flaky PMMA/Cu powders; SEM micrographs of triturated PMMA dispersed on the surface of (**c**) PMMA /Cu-1, (**d**) PMMA /Cu-2, and (e) PMMA /Cu-3, correspondingly.

The morphology of the original spherical Cu powders is displayed in Fig. 2(a), and the size of Cu powders is about 30–40 μm in diameter. After ball-milling, Cu powders are transferred into small Cu flakes. Figure 2(b) shows the SEM image of ball-milled Cu powders which have been deformed into flake-like shape with smooth surfaces and a thickness of ~1μm. Surface area of Cu matrix is greatly improved, providing a larger position for the adherence of PMMA. At the same time, PMMA powders are pulverized into smaller particles and loaded on the surfaces of the Cu flakes in the presence of mechanical force. Images of triturated PMMA dispersed on the surfaces of Cu flakes of PMMA/Cu-1, PMMA /Cu-2 and PMMA /Cu-3 are shown in Fig. 2(c–e), in which PMMA/Cu-2 shows the most homogeneous dispersion of PMMA on Cu flakes. With the increase of PMMA, size of PMMA particles loaded on Cu flakes grows. According to these results, too much PMMA may result in the agglomeration of PMMA on Cu powders, leading to a larger size of PMMA particles.

**Figure 3:**
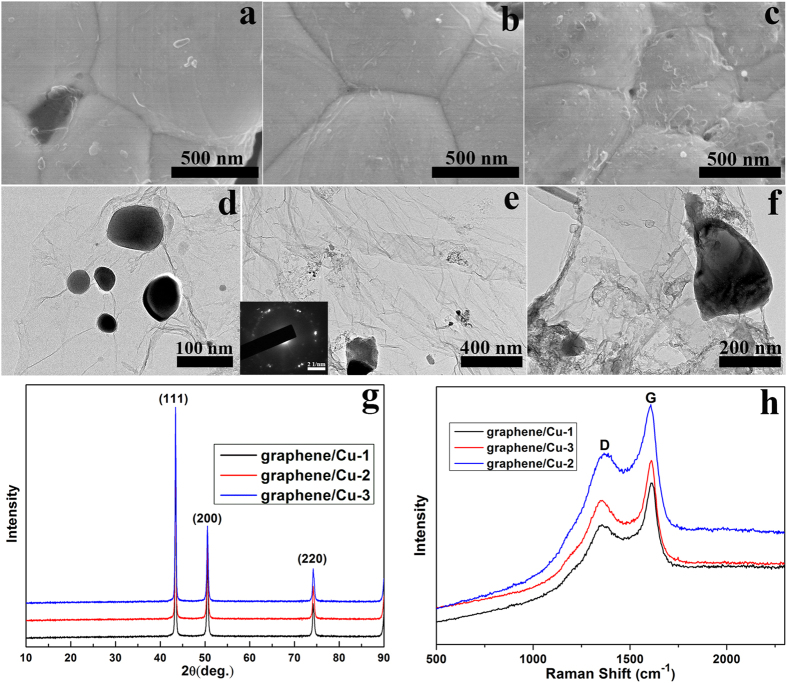
SEM morphologies of (a) graphene/Cu-1, (b) graphene/Cu-2 and (c) graphene/Cu-3, respectively; TEM morphologies of (d) graphene/Cu-1, (e) graphene/Cu-2 and (f) graphene/Cu-3, respectively. (g) XRD patterns of graphene/Cu-1, graphene/Cu-2 and graphene/Cu-3. (h) Raman spectrums of graphene/Cu-1, graphene/Cu-2 and graphene/Cu-3.

Figure 3(a–c) represent the morphologies of graphene *in-situ* grown on flaky Cu powders of graphene/Cu-1, graphene/Cu-2 and graphene/Cu-3, respectively. In graphene/Cu-1and graphene/Cu-2 composite powders, a whole piece of graphene covers several Cu grain boundaries and grain boundaries of the Cu matrix are clearly seen beneath embossed graphene, indicating the high light transmission and good crystallinity of *in-situ* graphene. Figure 3(e) provides a TEM image of the graphene showing a hexagonal selected area electron diffraction (SAED) pattern, which indicates clear the pattern of the few-layered graphene. In graphene/Cu-3 composite powders, SEM morphology of reduction products from PMMA is significantly different from those in graphene/Cu-1 and graphene/Cu-2 and only some decentralized graphene can be identified in the image.

To further explore the morphologies and quality of *in-situ* grown graphene in different composite powders, the Cu matrix is eliminated by using CuSO4 solution acidized with hydrochloric acid and graphene is observed by TEM. Morphologies of graphene from graphene/Cu-1, graphene/Cu-2, and graphene/Cu-3 are shown in Fig. 3(d–f). In Fig. 3(d,e), wrinkles of graphene are distinguishable in the images and the high transparency of the graphene results from its ultrathin structure. The size of graphene layer is up to micro level and areas with relatively larger contrast are residual Cu particles. Since graphene has a strong chemical resistance to the etching solution, Cu covered by graphene layer could not easily be totally etched^14^ and some residual nanoscale Cu particles can be seen in the TEM images. In Fig. 3(f), the product from PMMA does not own wavy edge structure and its light transmission is not so ideal in some areas. Judging from the characterizations of SEM, TEM and Raman, we suspect that the mass ratio of PMMA in PMMA/Cu-3 has beyond the ability of Cu to catalyze all PMMA into graphene and partial reduction products of PMMA/Cu-3 are thin carbon sheets instead of graphene.

Figure 3(g) shows the XRD results of graphene/Cu-1, graphene/Cu-2 and graphene/Cu-3 powders, respectively. Three diffraction peaks with high intensity correspond to the three crystalline planes of (111), (200) and (220) of the face centered cubic (fcc) Cu, respectively. No peaks of graphene are detected, because strong diffraction peaks of Cu matrix cover up information from graphene. Except for the peaks of Cu, no other peaks have been detected, indicating chemical stability between the Cu matrix and graphene. The Raman spectrums of three composite powders are shown in Fig. 3(h). The G band originates from the stretching motion of sp^2^ carbon pairs in both rings and chains, while the D band arises from defects in the hexagonal sp^2^ carbon network or the finite particle-size effect^23^. The relative intensity between the D and G peaks (I_D_/I_G_) reflects the quality of the CNTs or graphene and a higher ratio value may indicate a higher defect density^14^,^24^. I_D_/I_G_ ratios of graphene/Cu-1 and graphene/Cu-2 are measured about 0.76 which demonstrates that graphene obtained through this method is of good crystallinity and structural integrity. With the increment of PMMA, the I_D_/I_G_ ratio of graphene/Cu-3 increases to 0.9, revealing that defects in graphene increase with solid carbon source.

In traditional methods, ball-mill is employed to disperse RGO within the metal matrix. While in this work, graphene *in-situ* grows on Cu matrix after ball-mill of Cu powders and PMMA. In comparison, not only mechanical damage from ball-mill is totally avoided, morphology of graphene could also be kept its as original integrity when the bonding between graphene and Cu matrix forms.

**Figure 4:**
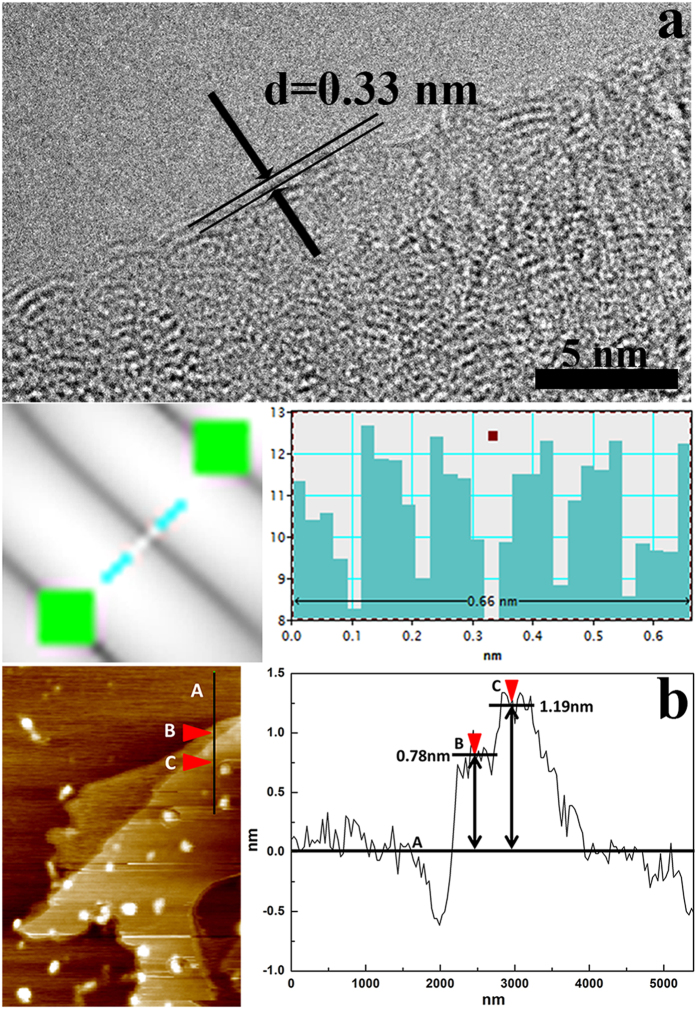
(**a**) HRTEM image of *in-situ* grown graphene and its interlayer space. (**b**) An AFM image and section analysis of *in-situ* grown graphene absorbed on freshly cleaved mica.

High-resolution TEM image of edges of graphene from graphene/Cu-2 is shown in Fig. 4(a) and about three layers of graphene can be identified. Interlayer space of graphene layers is measured through Fourier transform by using Digital Micrograph. Considering the deviations in measurement, a 0.66 nm interlayer space is obtained by measuring the space between three graphene layers after Fourier transform and inverse Fourier transform. Thus, the interlayer space of *in-situ* grown graphene is 0.33nm and it is quite close to the theoretical value (0.34 nm)^4^, proving the good quality of *in-situ* grown graphene. AFM is also an effective method to measure the thickness of the samples. Figure 4(b) shows a typical AFM image and section analysis of *in-situ* grown graphene from graphene/Cu-2 composite powders after the etching of Cu matrix. In the image, two pieces of graphene overlap and the thickness of them are obtained by measuring the thickness between B, C and A. The thicknesses of B and C areas are 0.78 nm and 0.41 nm, respectively. This means the two pieces of graphene are separately of three layers and two layers, which is consistent with the data in Fig. 4(a).

**Figure 5:**
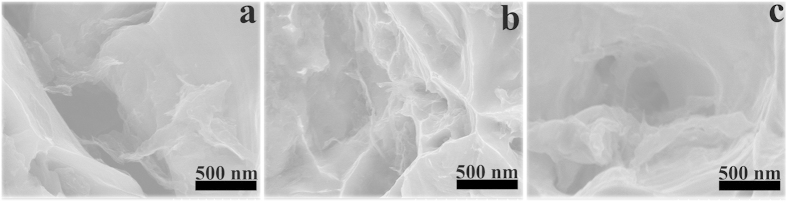
Fracture surfaces of (a) graphene/Cu-1, (b) graphene/Cu-2 and (c)graphene/Cu-3, correspondingly.

Figure 5 represents the fracture surfaces of graphene/Cu-1, graphene/Cu-2, and graphene/Cu-3. Among the dimples of fractures, ripped graphene could be clearly seen pulled out from the fracture surfaces of graphene/Cu-1 and graphene/Cu-2. Specially, *in-situ* grown graphene is homogeneously dispersed throughout the graphene/Cu-2 composite without agglomeration (Fig. 5(b)) because graphene has been *in-situ* grown on Cu flakes before the hot-press sintering process. However, in graphene/Cu-3 (Fig. 5(c)), thin carbon sheets could be identified in the dimples of its fracture surface. Thin carbon has been separated from the matrix and there is no obvious sign of adhesion between them.

**Figure 6:**
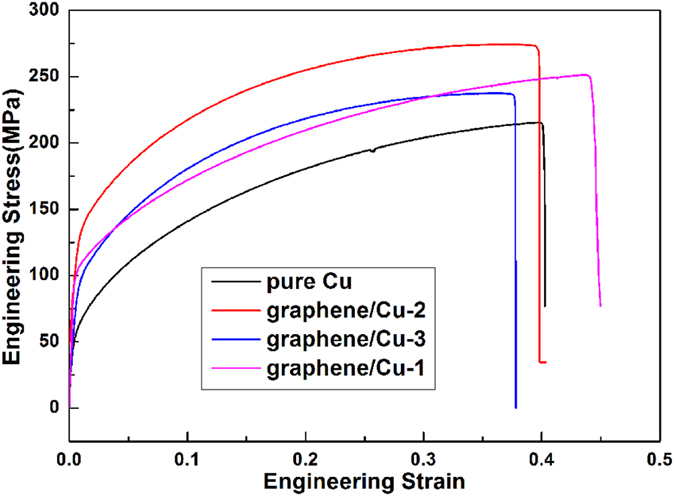
Stress–strain curves of pure Cu and different graphene/Cu composites.

Mechanical properties of the pure Cu and graphene/Cu composites are listed in Table 1 and Fig. 6 shows the stress-strain curves of graphene/Cu composites and pure Cu. It is obvious that there is a marked improvement on the mechanical properties of the graphene/Cu composites. Among the composites, graphene/Cu-2 exhibits a tensile strength of 274 MPa and yield strength of 244 MPa, which are respectively 27.4% and 177% higher than pure Cu. Strengthening effect in yield strength is more effective than the impregnation method (120%)^25^. Both yield strength and tensile increase with the increase of graphene content compared with graphene/Cu-1 composite. However, mechanical properties of graphene/Cu-3 fall to a quite low level. The poor enhancement of graphene/Cu-3 may primarily results in the thin carbon sheets as shown in Figs. 3(f) and 5(c), which is deleterious to the mechanical properties of composites. Graphene/Cu-3 also shows a significant decrease in elongation compared to other composites, further indicating the detrimental effect of thin carbon sheets within the composite. It is noteworthy to mention that graphene/Cu-1 shows an ameliorative toughness than pure Cu, and this result comes from the excellent modulus of graphene. This phenomenon reveals that the composite can be strengthened without the loss of good ductility with *in-situ* grown graphene. The hardness value of pure Cu is much higher than common Cu, which is the consequence of cold hardening of ball-milling process.

To get the precise contents of *in-situ* grown graphene within graphene/Cu composites, thermogravimetric tests are conducted to measure the mass fractions of composites. Results of thermogravimetric tests are summarized in Table 1. Graphene contents of composites are lower than design values, this is because carbon atoms derived from pyrolyzed PMMA can diffuse with the flowing atmosphere. We suspect that the airflow in the quartz tube takes away a part of C atoms during the catalyzing process, leaving particle carbon atoms catalyzed to graphene on the Cu matrix. Since graphene/Cu-3 contains many thin carbon sheets except graphene, it is no easy to get the accurate content of graphene in graphene/Cu-3.

The theoretical density value of the composite can be calculated from the equation (1):


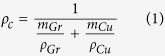


where, 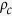, 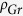 and 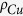 are densities of graphene/Cu composites, graphene and Cu matrix, respectively. 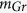 and 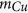 are mass fractions of graphene and Cu matrix. Density of graphene is estimated as 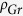 = 1.06 g/cm^3^ 26 and theoretical density value of graphene/Cu-2 composite (0.95 wt.% graphene) is 8.31 g/cm^3^. Density of graphene/Cu-2 composite is measured 8.28 g/cm^3^ according to the test, which is quite close to the theoretical density value. This shows that hot-press sintering procedure almost realizes complete densification of the composite.

Electricity conductivities of composites are very close to that of a standard annealed Cu conductor, 57.5 × 10^6^ S·m^−1^ of International Annealed Copper Standard (IACS)^25^. The electrical conductivities are evaluated by the eddy current method. The conductivity of pure Cu is measured to be 57.1 × 10^6^ S·m^−1^, and the conductivities of graphene/Cu-1 composites, graphene/Cu-2 composites and graphene/Cu-3 composites are measured to be 57.3 × 10^6^ S·m^−1^, 57.5 × 10^6^ S·m^−1^ and 56.4 × 10^6^ S·m^−1^, respectively. As we can see, electrical conductivities of graphene/Cu-1and graphene/Cu-2 are superior to pure Cu. Due to increment of defect in obtained *in-situ* grown graphene, electrical conductivity of graphene/Cu-3 is inferior to pure Cu and the other two graphene/Cu composites. The effect of graphene within the matrix is like “conductive films”, guaranteeing the transferring of current in composites. In this light, electricity conductivities of graphene/Cu composites enhanced by *in-situ* grown graphene keep at a satisfactory level for the usage in electronics.

## Discussion

So far, strengthening mechanism of MMCs can be elucidated by many theories. Load transfer^27^, dislocation strengthening^28^, solid-solution strengthening^29^, precipitation strengthening^30^ and grain refinement^31^ are generally considered to be the strengthening mechanisms in MMCs. Among these mechanisms, load transfer and dislocation strengthening make significant contributions to strengthening effects in graphene/metal composites^22^.

**Figure 7:**
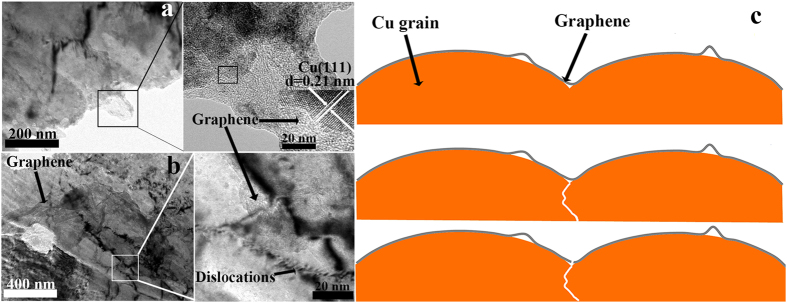
(**a**) TEM morphology of interface between graphene and Cu matrix in graphene/Cu-2 composite. (**b**) Dislocation strengthening effect of graphene within graphene/Cu-2 composite. (**c**) Schematic diagram of a sectional view of graphene/Cu in the fracture process.

Excellent interfacial between reinforcement and matrix is a key factor in guaranteeing strengthening effect in composites. With regard to a composite, many factors may affect the interface and interfacial bonding occupies a considerable impact. HRTEM is used to study the interface between graphene and the Cu matrix in graphene/Cu-2 composite. Since the solubility of C atoms in the Cu matrix is extremely low, graphene *in-situ* grown on Cu flakes is quite thin and forms well-contacted interface with the Cu matrix, which is shown in Fig. 7(a). The inset HRTEM image reveals a lattice spacing of 0.21 nm, which matches the (111) plane of cubic Cu. The (111) surface of Cu is able to catalyze PMMA into the highest quality of graphene in epitaxial growth, in terms of structural integrity, because of the close lattice match between hexagonal graphene (lattice constant 2.46 A° at 573 K) and hexagonal (111) Cu (lattice constant 2.56 A° at 573 K)^32^. The bonding between graphene and (111) Cu interface is of extremely high strength according to molecular dynamics simulations^33^. Graphene layers are clearly seen and there is no gap or impurity along the interfaces between the graphene and Cu matrix in the HRTEM image, indicating that interfacial bonding between *in-situ* grown graphene and Cu matrix is of high strength. The deformed graphene/Cu-2 composite after tensile test is further analyzed using TEM to gain insight into the strengthening mechanism. In Fig. 7(b), graphene is observed tightly adhered to the matrix. Structure integrity of graphene is kept intact after hot-press sintering and no aggregation of graphene happens. The dislocations are seen to pile up near graphene, and the density of dislocations is higher than that of other areas, indicating that graphene acts as obstacles to the propagation of dislocations during the deformation. Inset of Fig. 7b shows the graphene stretches across the region of dislocations.

Figure 7(c) is a schematic diagram of a sectional view of graphene/Cu. In consideration of the effect of the gas stream during the catalyzing process, a small proportion of C atoms may form convex graphene and most parts of the graphene layer is attached tightly to the Cu grains. Thus, a well-contacted interface and good bonding between Cu matrix and graphene guarantee the efficiency of load transfer during the deformation. When the composite is under stress, graphene sustains a certain part of load transferred from the matrix in the process of deformation. Since graphene has a much higher strength than Cu, Cu matrix fractures before than graphene. After the fracture of Cu matrix, graphene is lengthened and an extra force is needed to achieve the complete fracture of graphene, which can be corroborated by the morphology of the fracture surface (Fig. 5(b)). Graphene exists in deep dimples and is pulled out from the Cu matrix, indicating that load transfer plays an important part in the strengthening effect. This schematic diagram is also helpful to explain the improvement in elongation of graphene/Cu-1 composite. Since graphene is of high modulus, it can also improve the toughness of composites when impeding the progress of fracture.

According to the rule of mixture of composites (equation (2))


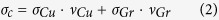


where 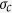, 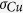 and 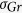 are tensile strengths of graphene/Cu composites, Cu matrix and graphene, 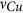 and 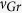 are volume fractions of Cu matrix and graphene.

In theory, graphene/Cu-2 composite actually should exhibit an outstanding improvement in mechanical properties. However, the actual increment in tensile strength is much less than theoretical value (4.47 GPa). This is mainly because of the different orientations of Cu flakes during the hot-press sintering process. As shown in Fig. 7(c), graphene on Cu flakes would not achieve a strengthening effect if the planes of the Cu flakes are vertical to instead of along the direction of load.

## Conclusions

In conclusion, graphene/Cu composite with structure-intact graphene uniformly dispersed within the Cu matrix has been successfully fabricated through *in-situ* growth of graphene on flaky Cu powders and vacuum hot-pressing. Graphene observed in the experiment mostly exists over Cu grain boundaries and forms a face-face bond with flaky Cu powders. The yield strength of 144 MPa and tensile strength of 274 MPa are achieved in graphene/Cu composite with 0.95 wt.% graphene, which are respectively a 177% and 27.4% enhancement over pure Cu. Strengthening effect of *in-situ* grown graphene in the matrix is contributed to load transfer and dislocation strengthening. Composites fabricated in this method can be strengthened as well as toughed with graphene *in-situ* grown within Cu matrix because of excellent modulus of graphene and high-strength interface. The novel fabrication method of graphene/Cu composite with *in-situ* grown graphene is meaningful to the design and mass production of MMCs. Optimization of the size of original Cu powders and the hot-press sintering temperature and this issue will be researched in future works.

## Methods

### Fabrication of PMMA/Cu composite powders

Initial materials include atomized Cu powders (99.9% purity, −400 meshes) and PMMA powders (99.9% purity, about 80 μm in diameter). The initial powders were produced by mixing 0.1, 0.2 or 0.3 g PMMA, 10 g Cu powders, and 150 g stainless steel balls and ball-milled at a speed of 400 rpm for 2 h with aRGOn (Ar) as a protective atmosphere. The composite powders prepared with the mass ratios between Cu and PMMA of 10:0.1, 10:0.2, and 10:0.3 were designated as PMMA/Cu-1, PMMA /Cu-2 and PMMA /Cu-3, respectively.

### Fabrication graphene/Cu composite powders and hot-press sintering

Then, the PMMA/Cu composite powders were put into a quartz tube furnace (preheated to 800 °C) and calcined for 10 min and rapidly cooled down to room temperature in the air. The whole calcination process was conducted under Ar (200 ml/min) and H_2_ (100ml/min) atmosphere. After calcination, the composite powders were later designated as graphene/Cu-1, graphene/Cu-2 and graphene/Cu-3, respectively. The graphene/Cu composite powders were then put into a graphite die and hot-pressed into ϕ45 × 3 mm^3^ samples under vacuum (below 10^−4^ MPa) using a pressure of 50 MPa at 800 °C for 1 h. For comparison, a pure Cu bulk sample was also fabricated through the same process.

### Microstructure and mechanical properties of the bulk composites

Morphologies of the pure Cu, PMMA/Cu and graphene/Cu powders were observed using a scanning electron microscope (SEM, HITACHI S4800). Thin foils for TEM observations were prepared by twin-jet electro-polishing at 100 mA in a solution of 30% nitric acid and 70% methanol solution cooled to −30 °C and observed on a JEM-2100F transmission electron microscope (TEM), JEM-2100F high-resolution transmission electron microscope was employed to characterize the morphology of the graphene. A micro-Raman spectrometer (Renishaw, inVia microscope) with a 532 nm laser was used to study the quality of graphene grown from PMMA. X-ray powder diffraction (XRD) patterns were recorded using a Rigaku D/max diffractometer with Cu Kα radiation at a wavelength of 1.5406 Å. Thermogravimetric analysis was carried out on a TGA 9000 thermogravimetric analyzer. Vickers hardness tests were conducted on an EveroneMH-6 machine. Tensile test samples were machined into specimens with the size of 17 × 5 × 2 mm3 and tensile tests were performed on a CSS-44100 electronic universal testing machine with 1 mm/min loading speed.

## Additional Information

**How to cite this article**: Chen, Y. *et al.* Fabrication of *in-situ* grown graphene reinforced Cu matrix composites. *Sci. Rep.* **6**, 19363; doi: 10.1038/srep19363 (2016).

## Figures and Tables

**Figure 1:**
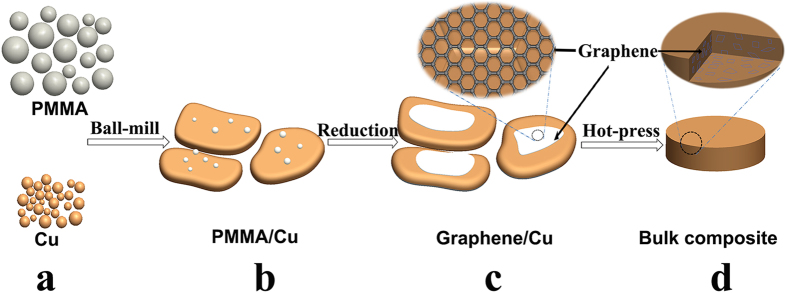
Schematic diagram of the fabrication procedures to fabricate graphene/Cu composites. **(a)** Original Cu powders and PMMA. **(b)** Flaky Cu powders loaded with PMMA after ball-milling. **(c)** Graphene/Cu composite powders. **(d)** Bulk graphene/Cu composite after hot-press sintering.

**Table 1:** Mechanical properties of pure Cu and different graphene/Cu composites.

Materials	Graphene content (wt.%)	HV	YS (MPa)	σ_UTS_(MPa)	ε_f_
Pure Cu	0	123	52	215	0.40
Graphene/Cu-1	0.4	131	103	251	0.44
Graphene/Cu-2	0.95	143	144	274	0.39
Graphene/Cu-3	—	135	98	238	0.37
